# Novel Visual Grade and Hounsfield Unit Predict Adequate Bone Strength for Cementless Total Knee Arthroplasty

**DOI:** 10.3390/medicina61112018

**Published:** 2025-11-12

**Authors:** Dong Hwan Lee, Dai-Soon Kwak, Sheen-Woo Lee, Yong Deok Kim, Nicole Cho, In Jun Koh

**Affiliations:** 1 Department of Orthopaedic Surgery, Yeouido St. Mary’s Hospital, Seoul 07345, Republic of Korea; ldh850606@naver.com; 2 Department of Orthopaedic Surgery, College of Medicine, The Catholic University of Korea, Seoul 06591, Republic of Korea; seraph622@naver.com; 3 Catholic Institute for Applied Anatomy, Department of Anatomy, College of Medicine, The Catholic University of Korea, Seoul 06591, Republic of Korea; daisoon@catholic.ac.kr; 4 Department of Radiology, Eunpyeong St. Mary’s Hospital, The Catholic University of Korea, Seoul 03312, Republic of Korea; leesw1@catholic.ac.kr; 5 Joint Replacement Center, Eunpyeong St. Mary’s Hospital, Seoul 03312, Republic of Korea; 6 Hackensack Meridian School of Medicine, 123 Metro Blvd, Nutley, NJ 07100, USA; nicole.cho@hmhn.org

**Keywords:** bone density, monitoring, intraoperative, tomography, X-ray computed, cementless, arthroplasty, replacement, knee

## Abstract

*Background and Objectives*: The use of cementless total knee arthroplasty (TKA) is increasing, but established methods for assessing bone quality to prevent early failure remain undefined. Current preoperative assessments using central bone mineral density (BMD) do not accurately reflect peripheral bone quality, and intraoperative evaluation is subjective. This study aimed to establish objective assessment methods by analyzing the correlations between a novel visual grading system, CT Hounsfield units (HU), and actual bone strength. *Materials and Methods*: This prospective study included 131 patients undergoing posterior-stabilized TKA. We developed a novel visual grading system (Excellent, Good, Fair, Poor) based on femoral cutting surface characteristics. CT HUs were measured preoperatively by an assisting surgeon in the box bone area. Femoral box specimens underwent indentation testing to determine their actual bone strength. Minimum Required Strength (MRS) was defined at 2.5-fold the patient’s body weight, and Estimated Withstanding Strength (EWS) was determined by scaling first failure load using area ratios. Patients were classified as “cementless suitable” (EWS > MRS) or “cemented mandatory” (EWS < MRS). Correlations were assessed using Spearman’s rank correlation for visual grade and Pearson correlation for Hounsfield units. ROC curve analysis determined diagnostic accuracy. *Results*: Visual grade exhibited an exceptionally robust relationship to bone strength (Spearman ρ = 0.903, *p* < 0.01), whereas HU showed substantial correlation (Pearson r = 0.660, *p* < 0.01, R^2^ = 0.435). Visual grading achieved excellent diagnostic accuracy (AUC = 0.974, sensitivity 95.1%, specificity 95.9%) using “Good” grade as cutoff. HU demonstrated AUC of 0.938 with 92.7% sensitivity and 81.6% specificity at a cutoff value of 65.2. *Conclusions*: Our novel visual grading system and CT HU demonstrated excellent correlations with actual distal femoral bone strength and outstanding diagnostic performance for identifying cementless TKA candidates. Unlike traditional subjective intraoperative assessments such as the “thumb test”, this system provides objective visual criteria directly correlated with actual bone strength. Preoperative HU screening with intraoperative visual grading can help prevent early failure.

## 1. Introduction

Recent studies have demonstrated an increasing proportion of total knee arthroplasty (TKA) procedures performed in younger patients [1,2]. The rise in young, active patients and growing obesity prevalence has contributed to evolving revision patterns, with aseptic loosening emerging as a predominant cause of implant failure [3,4,5]. Consequently, the utilization of cemented TKA has declined, while cementless TKA adoption has progressively increased [6,7]. This trend reflects technological advances including 3D printing, porous metal coating, and biological agents, which have improved fixation compared to earlier cementless implants [8,9]. Although multiple studies report favorable mid-term outcomes with promising long-term results anticipated, preventing early failure remains crucial for successful cementless TKA [10,11]. However, no consensus exists regarding optimal methods for assessing adequate bone quality, which is fundamental to achieving this goal.

Bone quality assessment most commonly relies on central bone mineral density (cBMD) measured by dual-energy X-ray absorptiometry (DEXA). While cBMD provides valuable information about bone density at the spine and femur, it does not directly or precisely reflect bone quality at peripheral joints [12,13,14]. Consequently, various studies have investigated direct peripheral joint bone quality assessment using modalities such as conventional computed tomography (CT), quantitative CT (QCT), and dual-energy CT (DECT) [15,16,17,18]. Among these modalities, conventional CT is frequently obtained for preoperative planning and represents readily available equipment in most hospitals, enhancing its clinical utility. Beyond these preoperative assessment methods, standardized intraoperative assessment techniques remain undefined. Growing evidence shows that bone quality cannot be reliably predicted by single preoperative parameters, emphasizing the need for multimodal and intraoperative assessment approaches [19,20]. Current intraoperative approaches rely on subjective methods such as the tactile sensation when pressing cancellous bone with fingers or the resistance felt during sawing and drilling procedures, which lack clear criteria and objective standards [8]. The authors developed a novel visual grading system as an intraoperative assessment method and examined the correlation of visual grade and actual bone strength, as well as between CT Hounsfield units (HU) and actual bone strength, to demonstrate that these two methods would be useful for evaluating cementless TKA suitability.

Therefore, we asked the following questions: (1) Do a novel intraoperative visual grading system and CT HU measurements of the distal femur correlate with actual bone strength? (2) How accurately can the visual grade and CT HU identify suitable candidates for cementless TKA?

## 2. Materials and Methods

### 2.1. Study Design and Setting

This prospective study included 131 patients who underwent posterior-stabilized TKA at our hospital between May 2022 and May 2024. All patients underwent preoperative knee CT one week before TKA and were treated with the Triathlon^®^ implant system (Stryker Inc., Mahwah, NJ, USA). Patients who did not provide informed consent were planned to be excluded; however, all patients provided consent.

### 2.2. Patient Demographics

The cohort consisted of 131 patients whose mean age was 68.4 ± 5.3 years (range, 53–86 years). Female patients represented 78% of the study population (102 patients), while male patients accounted for 22% (29 patients). Mean patient characteristics included height of 155.5 ± 7.3 cm (range, 143.1–177.2 cm), weight of 64.9 ± 9.0 kg (range, 45.4–91.5 kg), and body mass index of 26.8 ± 3.2 kg/m^2^ (range, 19.6–36.3 kg/m^2^). DEXA assessment revealed mean T-scores of −0.4 ± 1.6 for the lumbar spine and −1.2 ± 1.0 for the femoral neck, with normal bone density in 64% and 41%, osteopenia in 29% and 50%, and osteoporosis in 6% and 9% of patients at the lumbar spine and femoral neck, respectively (Table 1).

### 2.3. Hounsfiled Unit Measurement

**Figure 1:**
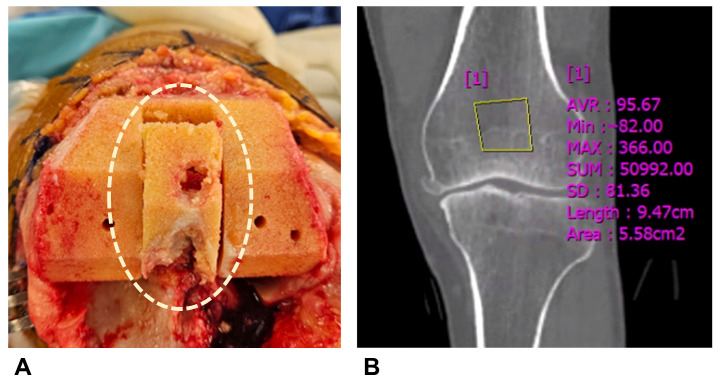
Hounsfield unit measurement. (**A**) Box bone area where Hounsfield unit was measured. (**B**) Hounsfield units were measured by setting rectangular regions of interest in the box bone area on CT coronal images.

Preoperative CT imaging was obtained one week before TKA on a CT scanner (SOMATOM Force; Siemens Healthineers, Erlangen, Germany). Images were obtained in supine position with craniocaudal direction with 1 mm slice thickness. HU measurements were performed on a single coronal slice at the level of the box region, using one rectangular region of interest (ROI) per site. The HU value obtained from this slice was used for each patient (Figure 1). Measurements were performed preoperatively by the assisting surgeon and exclusively conducted within cancellous bone regions of the box area, excluding cortical bone to minimize measurement error.

### 2.4. Definition of Visual Grading System

**Figure 2:**
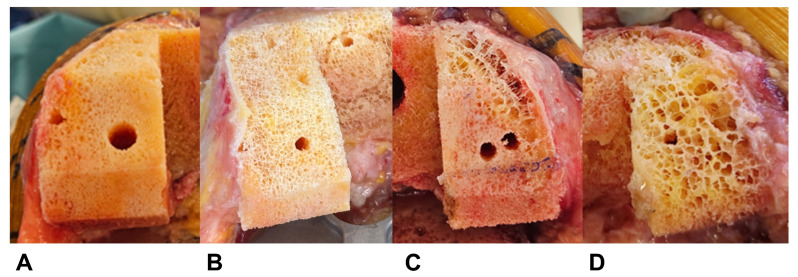
Representative examples of each visual grading scale. (**A**) Excellent, (**B**) Good, (**C**) Fair, and (**D**) Poor. The visual appearance of the femoral cutting surface after pulsed lavage was classified into a 4-point Likert scale based on pore characteristics and contour integrity (Table 2).

We established the visual grading system according to femoral cutting surface characteristics during TKA. Assessment was performed after pulsed lavage. We employed a 4-point Likert scale consisting of Excellent, Good, Fair, and Poor grades (Figure 2, Table 2). Our assessment focused on pore characteristics and contour integrity, with a 2 mm pore diameter as the dividing criterion. The 2 mm criterion was based on our indentation testing protocol, where maximum force application resulted in 1.8 mm displacement measurements. The detailed indentation testing procedure, including specimen preparation, probe specification, loading protocol, and data acquisition, is described in Section 2.6.

### 2.5. Reliability of Visual Grading System

Visual grading assessment was conducted independently by two orthopedic surgeons during surgery: the operating surgeon and the assisting surgeon, both of whom were fellowship-trained knee specialists with extensive surgical experience. We assessed inter-observer reliability using intraclass correlation coefficient (ICC). Visual grading system showed high reliability with an ICC of 0.91 (95% CI, 0.88–0.97).

### 2.6. Bone Strength Measurement

**Figure 3:**
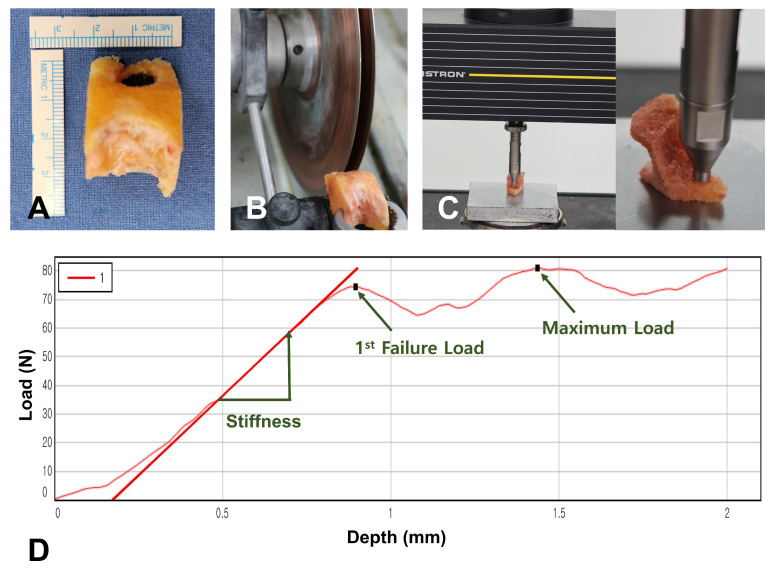
Indentation testing process. (**A**) Box bone specimen used for the experiment. (**B**) Box bone specimens were prepared to 6 mm thickness using a precision sectioning saw. (**C**) Indentation testing was performed using a 6 mm diameter flat-ended probe. (**D**) Force-displacement curve. First failure load was used as the primary measurement as it represents the onset of structural failure.

Mechanical testing was performed to directly measure bone strength using an established indentation protocol [13,21,22]. During femoral box preparation, bone specimens were collected and frozen at −70 °C within several hours for preservation. The day before testing, specimens were thawed to room temperature and prepared to uniform 6 mm thickness with a precision sectioning saw (IsoMet 5000; Buehler, Lake Bluff, IL, USA) using dual diamond blades. All mechanical tests were performed within one month after collection. An Instron machine (Instron 5567; Norwood, MA, USA) was used with a 6 mm diameter flat-ended probe (28.3 mm^2^ contact area). Testing commenced with 2N preload establishment, followed by compression at 1.0 mm/min rate to 2 mm depth. Data acquisition occurred at 30 Hz frequency with analysis via manufacturer software (Instron Bluehill v4.23). The primary measurement was first failure load, representing the earliest point of non-linear response in the force-displacement curve, indicating onset of structural failure (Figure 3). Results showed mean first failure load of 66.0 ± 42.5 N (range, 6.7–202.5 N), with displacement of 0.9 ± 0.3 mm, while maximum force was 85.6 ± 47.0 N at 1.8 ± 0.3 mm displacement (Table 3).

### 2.7. Definition of Mechanical Criteria for Cementless TKA Suitability

For determining cementless TKA eligibility, we defined two mechanical parameters. The Minimum Required Strength (MRS) was set at 2.5-fold the patient’s body weight, derived from biomechanical literature, indicating that routine postoperative activities produce force levels of this range at the knee [23,24,25]. For each specimen, the Estimated Withstanding Strength (EWS) was determined by scaling first failure load values according to the ratio of surface areas between the femoral component’s distal contact surface and our probe contact area (28.3 mm^2^) (Table 4). Patients were classified based on these metrics: those demonstrating EWS exceeding their individual MRS were designated “cementless suitable,” whereas those with EWS below their MRS were designated “cemented mandatory.” This classification framework facilitated evaluation of both visual grading and Hounsfield unit assessments as potential screening modalities for cementless TKA candidate selection.

### 2.8. Statistical Analysis

Statistical analysis was performed using SPSS software version 21 (IBM Corp., Armonk, NY, USA). We evaluated the relationship between visual grade with first failure load using Spearman’s rank correlation coefficient due to the ordinal nature of the visual grading system. The relationship between HU and first failure load was assessed through Pearson correlation analysis. To determine diagnostic accuracy of both assessment methods for identifying cementless TKA suitability, receiver operating characteristic (ROC) curve analysis was performed. We calculated area under the curve (AUC), sensitivity, specificity, positive predictive value (PPV), and negative predictive value (NPV) for each assessment tool, with optimal cutoff values determined for maximum diagnostic accuracy. For visual grading, each grade level (Excellent, Good, Fair, Poor) was evaluated as a potential diagnostic threshold to identify the most accurate cutoff for predicting adequate bone strength for cementless fixation. A value of *p* < 0.05 was considered statistically significant. Although a formal a priori sample size calculation was not performed, a post hoc analysis was conducted to confirm the adequacy of the sample size. Based on our observed event rate of 69% (91 of 131 patients classified as cementless suitable), an expected AUC of 0.90 (visual grading system), a null hypothesis AUC of 0.70, an alpha level of 0.05, and a power of 0.80, the minimum required sample size was 89 patients. Similarly, for the HU-based analysis (expected AUC 0.94), the required sample size was 67. Our final sample size of 131 exceeded both thresholds, indicating sufficient power for the diagnostic accuracy analyses.

## 3. Results

### 3.1. Correlations of Visual Grade and Hounsfield Units with Actual Bone Strength

**Figure 4:**
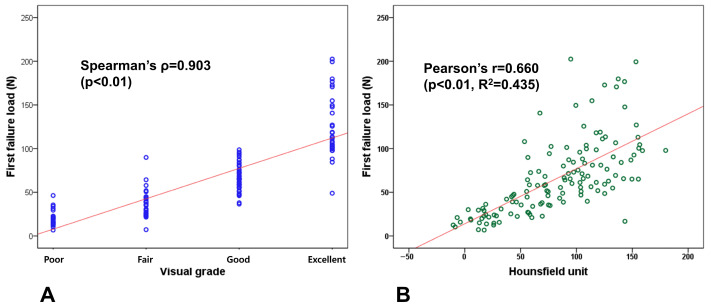
Correlation analysis with first failure load. (**A**) Visual grade shows strong correlation with a Spearman correlation coefficient (ρ) of 0.903 (*p* < 0.01). (**B**) Hounsfield units show substantial correlation with a Pearson correlation coefficient of 0.660 (*p* < 0.01).

Both visual grade and HUs demonstrated strong correlations with first failure load. The visual grading system showed a strong relationship with first failure load, achieving a Spearman correlation coefficient (ρ) of 0.903 (*p* < 0.01) (Figure 4A). Similarly, Hounsfield unit measurements demonstrated a substantial correlation with bone strength, yielding a Pearson correlation coefficient of 0.660 (*p* < 0.01) and an R^2^ value of 0.435 (Figure 4B). Additionally, both HU and first failure load increased progressively with higher visual grades from poor to excellent, demonstrating the consistency between visual appearance and bone strength (Table 5).

### 3.2. Diagnostic Performance of Visual Grade and Hounsfield Units for Cementless TKA Suitability

**Figure 5:**
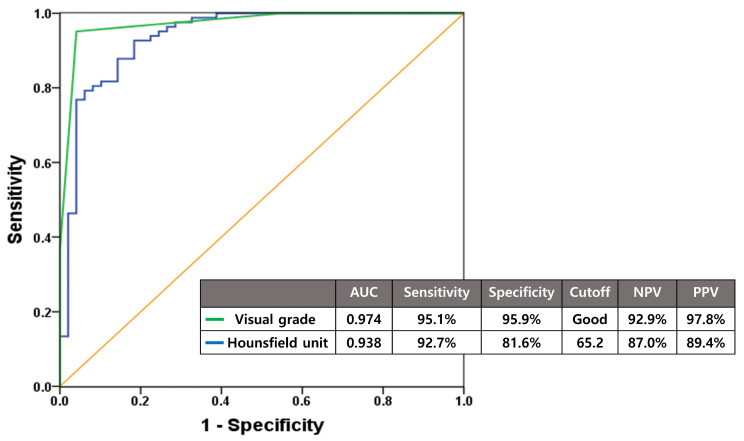
Diagnostic accuracy of visual grade and Hounsfield units assessed by ROC curve analysis for predicting cementless TKA suitability. With cutoffs set at Good grade and 65.2, respectively, both methods demonstrated excellent diagnostic accuracy for cementless TKA candidate selection.

Both visual grade and HU demonstrated excellent diagnostic accuracy for identifying suitable candidates for cementless TKA with minimal false-positive and false-negative rates. ROC curve analysis revealed that using “Good” visual grade as the cutoff yielded an AUC of 0.974 with 95.1% sensitivity, 95.9% specificity, 97.8% PPV, and 92.9% NPV. Hounsfield unit measurements achieved an AUC of 0.938 with 92.7% sensitivity, 81.6% specificity, 89.4% PPV, and 87.0% NPV when using a cutoff value of 65.2 (Figure 5).

## 4. Discussion

Cementless TKA has gained increasing attention owing to advances in implant technology and shifting patient demographics; however, early failure remains a clinical concern. Although adequate bone quality is essential to avoid early failure, no gold standard currently exists for evaluating bone quality specific to cementless TKA, underscoring the need for reliable preoperative and intraoperative assessment tools. We therefore developed a novel visual grading system to assess bone strength of the distal femur. We examined the correlations of preoperative and intraoperative assessments to actual bone strength, and we assessed the diagnostic accuracy of CT-derived HU and the visual grading system. Our findings demonstrated that both assessment methods exhibited strong relationships to actual bone strength and provided excellent diagnostic accuracy in determining cementless TKA candidacy.

Both our novel visual grading system and HU showed strong relationships with actual bone strength. In this study, the visual grading system achieved a Spearman correlation coefficient (ρ) of 0.903 (*p* < 0.01), while HU had a Pearson correlation coefficient of 0.660 (*p* < 0.01) with first failure load. Previous intraoperative assessment methods have relied on subjective approaches such as the thumb test, where surgeons press cancellous bone with their thumb, or evaluate haptic feedback during sawing, drilling, and keeling based on perceived resistance [26,27]. These existing methods depend on surgeon judgment and lack established correlations with actual bone quality. In contrast, our study established criteria based on displacement observed during maximum force application in indentation testing and showed close correlation with actual bone strength, providing more definitive evidence for bone quality assessment. HUs have been widely reported to correlate with bone mineral density, and similar correlations have been documented around the knee joint [15,28]. However, limited research has examined the correlation of HU to actual bone strength, especially for distal femur. Our study demonstrated a direct relationship with actual distal femoral bone strength, providing valuable evidence for clinical application. Our findings, together with previous studies, suggest that preoperative CT HU and the intraoperative visual grading system may serve as useful predictors of distal femoral bone quality in patients undergoing TKA.

Both our novel visual grading system and HU demonstrated excellent diagnostic accuracy in determining cementless TKA candidacy. When “Good” grade was set as the threshold, the visual grading system demonstrated an AUC of 0.974 (sensitivity 95.1%, specificity 95.9%) on ROC curve analysis, whereas HU showed an AUC of 0.938 (sensitivity 92.7%, specificity 81.6%) at a threshold of 65.2. These results indicate low rates of both false positives and false negatives, making both methods highly reliable for cementless TKA candidate selection. Across the visual grades, the “Good” grade demonstrated the highest AUC and provided the most balanced diagnostic performance in terms of sensitivity and specificity, supporting its role as the optimal cut-off for identifying sufficient bone quality for cementless fixation. This selection also reflects a conservative approach that minimizes the risk of inadequate fixation in borderline cases. Previous intraoperative assessment methods lack clear criteria and depend on subjective judgment [27,29]. Our novel visual grading system provides clear visual criteria, enabling more objective and consistent evaluation compared to these subjective approaches. Central BMD has been shown to inadequately reflect peripheral joint bone quality, leading to the development of various imaging modalities for peripheral BMD assessment, including QCT and DECT, which can provide specific bone quality evaluation [16,18,30]. However, these examinations require complex calculations using specialized software, expensive equipment, and skilled personnel [31,32,33]. Our results, taken together with previous studies, suggested that simple HU measurements from conventional CT scans, which are available in most hospitals, can provide adequate bone quality assessment with sufficient diagnostic accuracy for cementless TKA selection, offering high clinical utility.

This study had several limitations. First, our patient cohort was demographically homogeneous, comprising 78% females and only Asian patients. The need for bone specimens limited inclusion to TKA patients, creating an unavoidable selection constraint [34]. Future studies with more diverse populations including various ethnicities and balanced gender representation are needed for widespread application [35]. Second, our study was confined to single TKA implant. The specific box geometry and surface area dimensions of this implant directly affected our EWS calculations. Different manufacturers utilize distinct design features that may produce varying outcomes when our assessment methods are applied. To broaden the clinical applicability of this technique, validation studies across multiple implant designs will be necessary, possibly requiring manufacturer-specific cutoff values for optimal cementless TKA selection. Third, we utilized femoral box bone for indentation testing while employing the femoral component’s distal cutting area for EWS calculations due to consistent bone specimen collection feasibility. Since the box bone exhibits lower mechanical strength than the bone at the distal resection site, the EWS provides a conservative estimate that should not compromise cementless TKA success. However, these findings should be applied cautiously given the study’s inherent limitations. Additionally, this approach does not reflect tibial bone strength, which presents a clear limitation since actual subsidence occurs more frequently in the tibia. Tibial bone specimens were difficult to obtain with sufficient quality and consistency for experimental purposes. However, without tibial side assessment, our experimental approach cannot definitively confirm overall cementless implant suitability, thereby substantially limiting the clinical applicability of our findings. Therefore, future studies must overcome the technical challenges of tibial bone specimen acquisition and establish validated assessment methods for tibial bone quality to complete the comprehensive evaluation framework for cementless TKA. Fourth, visual assessment was conducted following pulsed lavage of the cutting surface. Alternative irrigation techniques, such as bulb syringe methods, may alter surface appearance and influence grading outcomes. Nevertheless, pulsed lavage delivers superior irrigation pressure compared to other approaches, minimizing potential impact on our visual grading system’s diagnostic reliability [36]. Fifth, potential measurement variability may exist in ROI delineation for box bone assessment. However, the rectangular configuration of the box bone facilitates straightforward ROI definition, and therefore measurement variability was deemed insignificant given this anatomical geometry. Sixth, Hounsfield unit values may vary for the same bone quality across different CT scanner models and settings. Scanner-specific calibration adjustments could address this issue and enable clinical implementation without compromising diagnostic utility. Finally, our mechanical criteria utilized simplified parameters, with MRS defined as 2.5-fold body weight and EWS based solely on femoral component distal surface area. These calculations do not account for individual variations in patient activity levels or surgical variables such as alignment and joint stability that may influence force distribution at the implant-bone interface. Furthermore, our experimental approach captured only axial compression resistance through indentation testing, whereas actual fixation failure mechanisms in cementless implants involve multiple directional forces—including interface micromotion, shear stresses, bending moments, and rotational loads—that are critical determinants of long-term stability. The inability to capture these multidirectional biomechanical factors represents a fundamental methodological constraint when evaluating comprehensive cementless TKA suitability. Future investigations incorporating these additional factors will be necessary to improve candidate selection accuracy for cementless TKA. Despite these limitations, our study successfully demonstrated that both the novel visual grading system and CT Hounsfield units serve as valuable tools for identifying appropriate cementless TKA candidates. Further research incorporating tibial bone assessment and multidirectional biomechanical factors should enhance selection accuracy and clinical applicability.

## 5. Conclusions

Our novel visual grading system and CT-derived HU demonstrated strong correlations with actual distal femoral bone strength and excellent diagnostic performance in identifying candidates for cementless TKA. The combination of preoperative CT HU measurements for screening and intraoperative visual grading for final decision-making may help reduce the risk of early failure after cementless TKA.

## Figures and Tables

**Table 1:** Baseline patient characteristics and osteoporosis status *.

Characteristics	Values (n = 131)	
Age (year)	68.4 ± 5.3 (53 to 86)	
Sex (women)	102 (78)	
Height (cm)	155.5 ± 7.3 (143.1 to 177.2)	
Weight (kg)	64.9 ± 9.0 (45.4 to 91.5)	
BMI (kg/m^2^)	26.8 ± 3.2 (19.6 to 36.3)	
Osteoporosis status		
	Lumbar spine	Femur neck
DEXA (T score)	−0.4 ± 1.6 (−3.6 to 4.9)	−1.2 ± 1.0 (−3.5 to 2.5)
Number of patients †		
Normal (T score > −1.0)	84 (64)	53 (41)
Osteopenia (−1.0 ≤ T score ≤ −2.5)	38 (29)	65 (50)
Osteoporosis (T score < −2.5)	9 (6)	13 (9)

* Values are reported as mean ± SD (minimum to maximum), with gender shown as female patient count and percentage in parentheses; † Values are reported as patient counts (percentage); BMI = body mass index; DEXA = Dual-energy X-ray absorptiometry.

**Table 2:** Definition of novel visual grading system.

Grade	Definition
Excellent	The resected bone surface exhibits a well-preserved cortical and trabecular architecture with rare or minimal porous defects. The contour of the resected surface remains intact and sharply defined, indicating optimal bone integrity suitable for cementless fixation.
Good	The surface shows multiple pores of <2 mm in diameter scattered across the resected area. Despite the presence of these small defects, the overall bony contour is well maintained, suggesting acceptable quality for mechanical interlocking.
Fair	The bone surface displays frequent porous defects > 2 mm. While the general contour of the resected area is preserved, the presence of large and numerous pores raises concerns about consistent implant support across the surface.
Poor	The surface is characterized by widespread porous defects > 2 mm in size, accompanied by clear collapse of the intended resection contour. This reflects compromised bone quality, potentially unsuitable for cementless fixation.

**Table 3:** Biomechanical properties of the box bone specimens *.

Parameters (n = 131)	Mean ± SD	Range (min–max)
First failure force (N)	66.0 ± 42.5	6.7~202.5
Displacement at first failure (mm)	0.9 ± 0.3	0.3~1.8
Maximal force (N)	85.6 ± 47.0	14.6~244.0
Displacement at maximal failure (mm)	1.8 ± 0.3	0.4~2.0
Stiffness (N/mm)	119.4 ± 88.6	12.0~533.8

* All values represent mean ± standard deviation.

**Table 4:** Component dimensions and the area ratio to the probe.

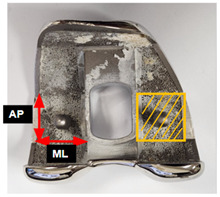	Size *	**Patient** **Number (%)**	**AP** **(mm)**	**ML** **(mm)**	**Area × 2** **(mm^2^)**	**Area** **Ratio**
	1	6 (5)	16.8	21.4	719	25.4
	2	20 (15)	18.1	22.9	829	29.3
	3	51 (39)	19.3	24.4	942	33.3
	4	35 (27)	19.3	25.9	998	35.3
	5	13 (10)	20.2	27.4	1107	39.1
	6	6 (5)	22.3	28.9	1289	45.5

* Size number of femoral component; AP = anteroposterior; ML = mediolateral.

**Table 5:** Mean Hounsfield unit (HU) and first failure load across visual grades *.

Visual Grade	HU	First Failure Load (N)
Poor	27.6 ± 33.3	21.6 ± 10.0
Fair	50.8 ± 29.1	33.6 ± 10.6
Good	104.3 ± 30.4	67.7 ± 15.5
Excellent	115.7 ± 29.5	127.5 ± 34.7

* All values represent mean ± standard deviation.

## Data Availability

The data that support the findings of this study are available from the corresponding author upon reasonable request.

## Abbreviations

The following abbreviations are used in this manuscript:

AP	Anteroposterior
AUC	Area under the curve
BMD	Bone mineral density
BMI	Body mass index
cBMD	Central bone mineral density
CT	Computed tomography
DECT	Dual-energy computed tomography
DEXA	Dual-energy X-ray absorptiometry
EWS	Estimated withstanding strength
HU	Hounsfield unit
ICC	Intraclass correlation coefficient
ML	Mediolateral
MRS	Minimum required strength
QCT	Quantitative computed tomography
ROC	Receiver operating characteristic
ROI	Region of interest
TKA	Total knee arthroplasty
